# Management of Severe Subcutaneous Emphysema With a Subcutaneous Cannula: A Life-Saving Approach

**DOI:** 10.7759/cureus.75865

**Published:** 2024-12-17

**Authors:** Chang Wai Lim, Leng Cheng Sia

**Affiliations:** 1 Department of Medicine, Universiti Malaya Medical Centre, Kuala Lumpur, MYS; 2 Division of Respiratory Medicine, Universiti Malaya Medical Centre, Kuala Lumpur, MYS

**Keywords:** catheter, chest tube, subcutaneous cannula, subcutaneous emphysema, therapy

## Abstract

Subcutaneous emphysema is a well-known complication of chest tube insertion that can become life-threatening. Severe cases often progress rapidly, necessitating prompt intervention to prevent complications such as airway obstruction and respiratory failure. We report the case of a 57-year-old man who developed extensive subcutaneous emphysema following chest tube insertion. Despite the reinsertion of the chest tube, the patient's symptoms worsened, prompting the use of a subcutaneous cannula. Two 14G subcutaneous cannulas were placed in the anterior chest wall, resulting in rapid decompression and significant symptom relief. The subcutaneous cannula technique is a simple, cost-effective, and minimally invasive method for managing severe subcutaneous emphysema. This approach offers a viable alternative to more invasive techniques, with the potential for rapid symptom resolution and reduced complications.

## Introduction

Subcutaneous emphysema is a recognized complication of tube thoracostomy that can progress rapidly and become potentially life-threatening. It may result in extensive air tracking across multiple tissue planes, causing significant discomfort, airway obstruction, cardiac tamponade, and tension pneumomediastinum. While no specific guidelines exist for the management of subcutaneous emphysema, various approaches have been described in the literature, including the use of intercostal drain on suction, wider-bore intercostal drains, and "blow-hole" incisions [1,2].

## Case presentation

A 57-year-old male with severe chronic obstructive pulmonary disease (COPD), classified as Global Initiative for Chronic Obstructive Lung Disease (GOLD) Group E, presented to the emergency department with difficulty in breathing for two days duration. Upon examination, he was tachypneic and sitting in a tripod position. His vital signs included a temperature of 36.8°C, blood pressure of 134/82 mmHg, heart rate of 120 beats per minute, respiratory rate of 38 breaths per minute, and oxygen saturation of 80% under room air. Chest auscultation revealed diminished breath sounds on the right side. Cardiovascular and other systemic examinations were unremarkable.

The patient received salbutamol nebulization and supplemental oxygen through a Venturi mask. However, the patient became more tachypneic and subsequently was intubated due to type 2 respiratory failure. A chest radiograph revealed a right-sided pneumothorax, necessitating the insertion of a right-sided tube thoracostomy. The patient was extubated on day 3 of admission, and a repeat chest radiograph showed complete re-expansion of the right lung. Subsequently, the chest tube was removed on the same day.

**Figure 1:**
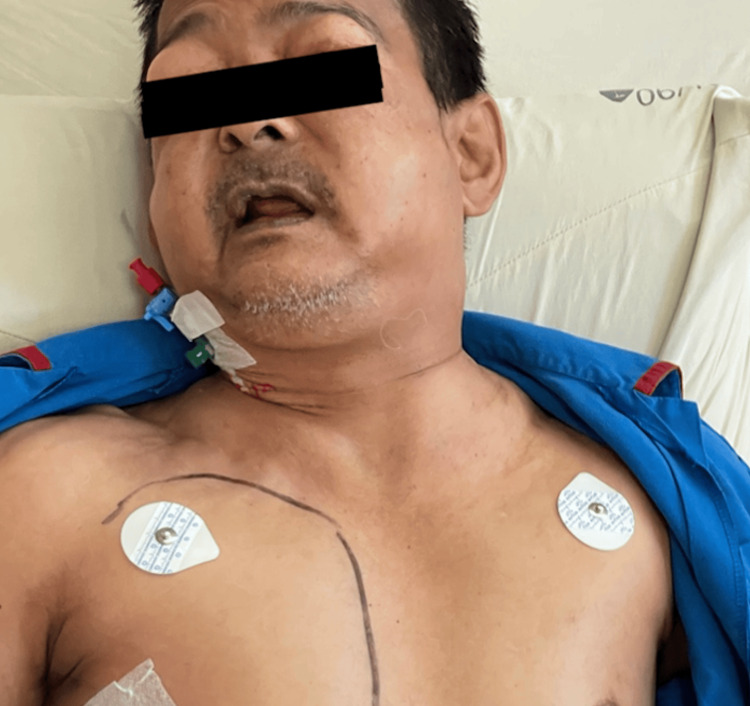
Clinical photograph of the patient showing extensive subcutaneous emphysema, causing closure of the palpebral fissures

**Figure 2:**
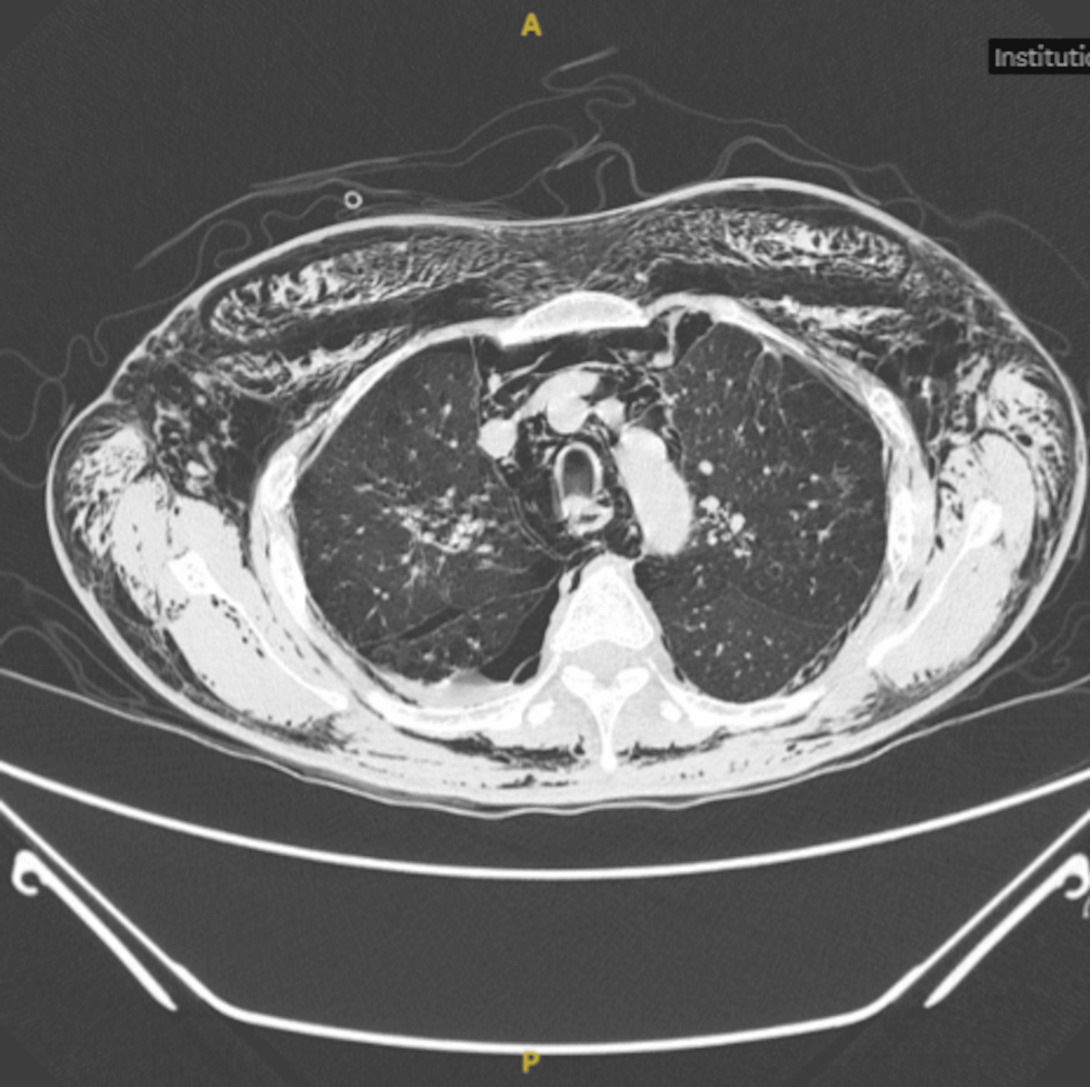
Computed tomography of the thorax (axial view) shows severe bilateral subcutaneous emphysema and pneumomediastinum

However, the following day, the patient experienced recurrent dyspnea accompanied by facial and neck swelling (Figure 1). Chest auscultation revealed reduced air entry over the right lung with hyperresonance on percussion. A 20 Fr right-sided chest thoracostomy was promptly reinserted. Contrast-enhanced computed tomography (CT) of the thorax (Figure 2) demonstrated extensive subcutaneous emphysema with pneumomediastinum and a small right-sided pneumothorax. Despite the presence of the chest tube thoracostomy, the subcutaneous emphysema worsened. Palpable crepitus was noted around the neck, and significant periorbital swelling, causing bilateral eyelid closure. He also complained of dysphonia and dysphagia. Based on the classification described by Aghajanzadeh et al, [3], this was a grade V subcutaneous emphysema.

With the patient’s symptoms worsening and distress increasing, subcutaneous cannulas were inserted. Two 14G cannulas were inserted subcutaneously into the anterior chest wall, one on the left and one on the right, both along the mid-clavicular line at a 45° angle, at the second intercostal space, with the tips positioned approximately 1 cm beneath the skin surface.

**Figure 3:**
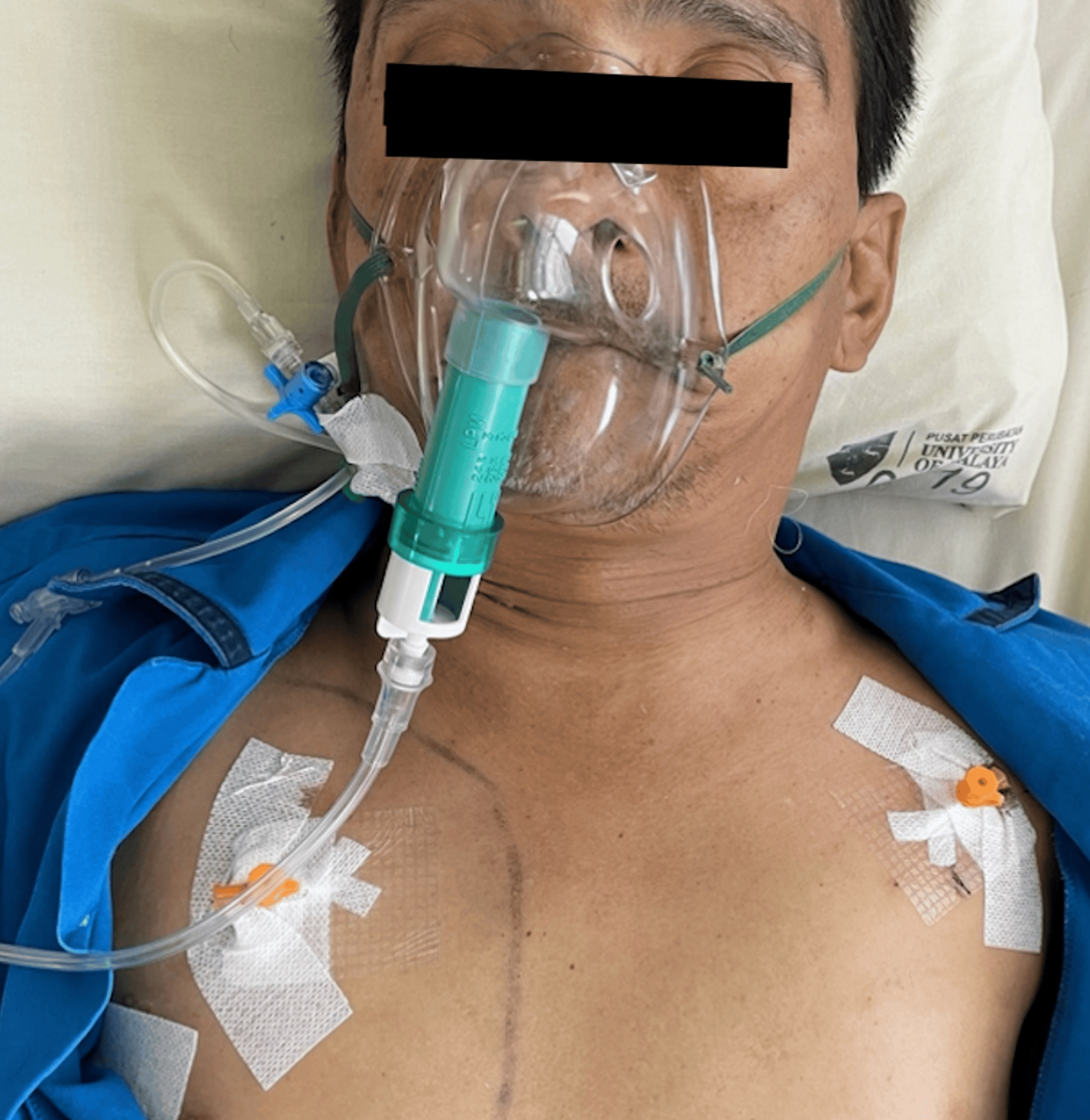
Clinical photograph of the patient 24 hours after the insertion of subcutaneous cannulas

**Figure 4:**
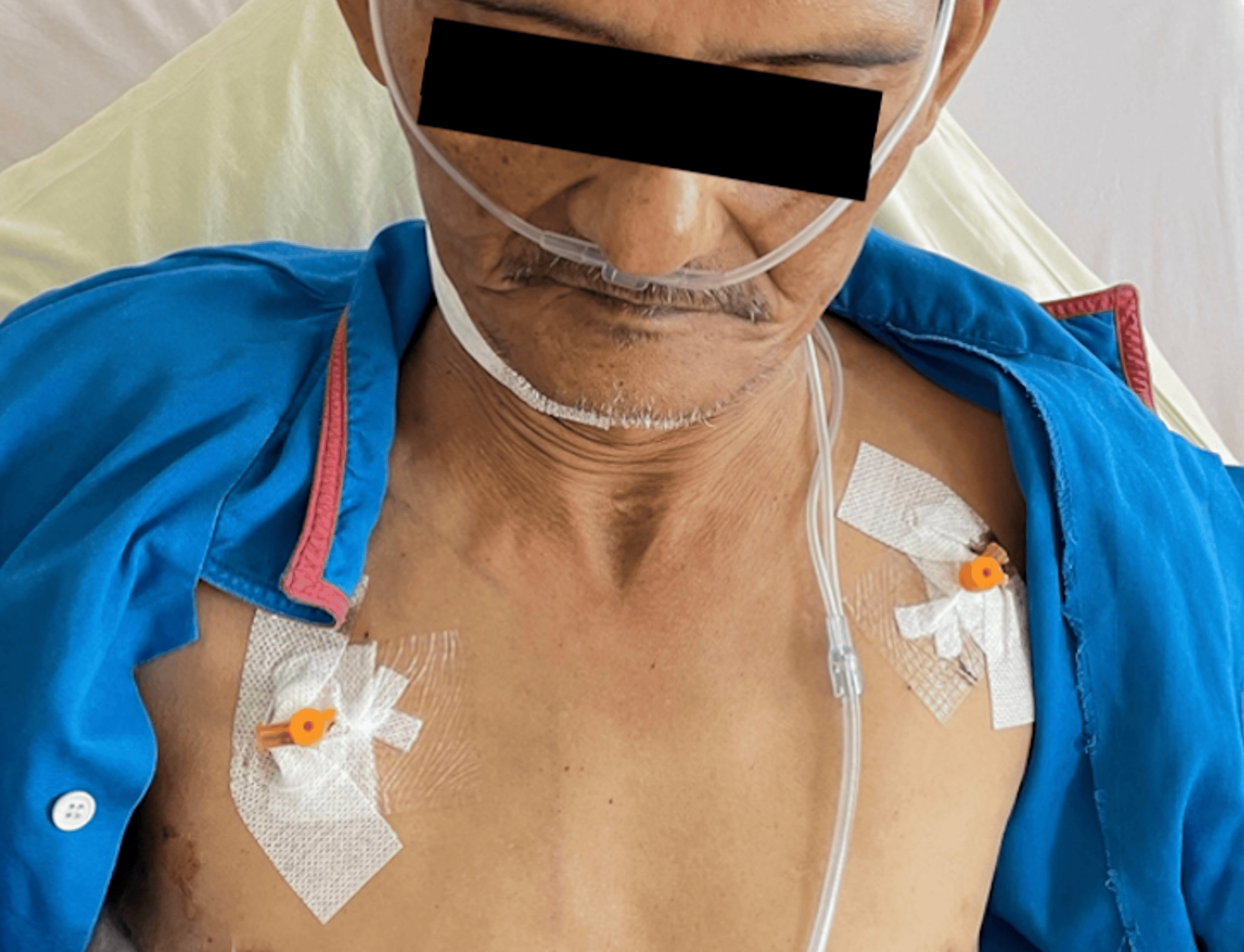
Clinical photograph of the patient three days after the insertion of subcutaneous cannulas, with the cannulas in situ

Following insertion, a “hissing” sound was heard, indicating the release of trapped air. Rapid decompression was achieved within two hours, after which the patient was able to open his eyes (Figure 3). The subcutaneous cannulas were secured with Tegaderm dressing and left uncapped. Oxygen supplementation was gradually reduced, and lung re-expansion was achieved after three days. The subcutaneous emphysema resolved (Figure 4), and the subcutaneous cannulas were then removed.

## Discussion

Subcutaneous emphysema can occur as a result of pneumothorax or as a complication following chest tube insertion. The causes of subcutaneous emphysema following chest tube insertion include tube blockage, improper tube placement, migration of the side port, and the use of multiple chest tubes [4]. When the parietal pleura is breached, air from a pneumothorax may escape into the chest wall and surrounding subcutaneous tissue [5]. Due to the interconnectedness of the visceral spaces between the neck, mediastinum, and retroperitoneum, air can spread to these areas or pass superficially through the endothoracic fascia into the subcutaneous tissues [6]. Air from the neck can also travel to the face and eyelids, potentially leading to palpebral closure, as observed in this case.

Subcutaneous emphysema is usually self-limiting and resolves spontaneously without any intervention. However, rapid expansion of subcutaneous emphysema can be life-threatening, leading to upper airway obstruction and respiratory failure [7,8]. Therefore, the primary goal in managing severe subcutaneous emphysema is to decompress the thoracic inlet and neck to maintain the airway. Various techniques have been reported for treating extensive subcutaneous emphysema, ranging from minimally invasive methods, such as subcutaneous thoracotomy tubes, to more invasive procedures like "blow hole" incision [2], and Penrose drain [1,9].

In this case, the subcutaneous cannulas proved effective in releasing air trapped under the skin, facilitating decompression and relieving subfascial pressure. This method offers several advantages as it does not require specialized equipment or open infraclavicular incisions, which can be associated with complications such as bleeding, inadequate depth, and poor cosmetic results. The subcutaneous emphysema in this case progressed rapidly, and the subcutaneous cannula was easily placed within minutes, in contrast to other methods. Beck et al. [10] and Perkins et al. [11] also described similar methods, which were proven successful in resolving subcutaneous emphysema within three days.

## Conclusions

The use of a subcutaneous cannula is an effective, well-tolerated, and minimally invasive technique for managing subcutaneous emphysema. Its simplicity, safety, and cost-effectiveness make it a practical option, particularly in resource-limited or urgent settings. This technique offers rapid symptom relief while minimizing patient discomfort and potential complications. This case highlights the use of a subcutaneous cannula as a simple, yet impactful approach to the treatment of subcutaneous emphysema.
